# Incretins and microvascular complications of diabetes: neuropathy, nephropathy, retinopathy and microangiopathy

**DOI:** 10.1007/s00125-023-05988-3

**Published:** 2023-08-19

**Authors:** Jonathan Goldney, Jack A. Sargeant, Melanie J. Davies

**Affiliations:** 1 Diabetes Research Centre, College of Life Sciences, University of Leicester, Leicester, UK; 2 NIHR Leicester Biomedical Research Centre, University Hospitals of Leicester NHS Trust and University of Leicester, Leicester, UK; 3 Leicester Diabetes Centre, University Hospitals of Leicester NHS Trust, Leicester, UK

**Keywords:** DPP-4 inhibitors, GLP-1, GLP-1 receptor agonists, Incretin, Mechanisms, Microvascular disease, Nephropathy, Neuropathy, Pathophysiology, Retinopathy, Review, Type 2 diabetes

## Abstract

Glucagon-like peptide-1 receptor agonists (GLP-1RAs, incretin mimetics) and dipeptidyl peptidase-4 inhibitors (DPP-4is, incretin enhancers) are glucose-lowering therapies with proven cardiovascular safety, but their effect on microvascular disease is not fully understood. Both therapies increase GLP-1 receptor agonism, which is associated with attenuation of numerous pathological processes that may lead to microvascular benefits, including decreased reactive oxygen species (ROS) production, decreased inflammation and improved vascular function. DPP-4is also increase stromal cell-derived factor-1 (SDF-1), which is associated with neovascularisation and tissue repair. Rodent studies demonstrate several benefits of these agents in the prevention or reversal of nephropathy, retinopathy and neuropathy, but evidence from human populations is less clear. For nephropathy risk in human clinical trials, meta-analyses demonstrate that GLP-1RAs reduce the risk of a composite renal outcome (doubling of serum creatinine, eGFR reduction of 30%, end-stage renal disease or renal death), whereas the benefits of DPP-4is appear to be limited to reductions in the risk of albuminuria. The relationship between GLP-1RAs and retinopathy is less clear. Many large trials and meta-analyses show no effect, but an observed increase in the risk of retinopathy complications with semaglutide therapy (a GLP-1RA) in the SUSTAIN-6 trial warrants caution, particularly in individuals with baseline retinopathy. Similarly, DPP-4is are associated with increased retinopathy risk in both trials and meta-analysis. The association between GLP-1RAs and peripheral neuropathy is unclear due to little trial evidence. For DPP-4is, one trial and several observational studies show a reduced risk of peripheral neuropathy, with others reporting no effect. Evidence in other less-established microvascular outcomes, such as microvascular angina, cerebral small vessel disease, skeletal muscle microvascular disease and autonomic neuropathies (e.g. cardiac autonomic neuropathy, gastroparesis, erectile dysfunction), is sparse. In conclusion, GLP-1RAs are protective against nephropathy, whereas DPP-4is are protective against albuminuria and potentially peripheral neuropathy. Caution is advised with DPP-4is and semaglutide, particularly for patients with background retinopathy, due to increased risk of retinopathy. Well-designed trials powered for microvascular outcomes are needed to clarify associations of incretin therapies and microvascular diseases.

**Graphical Abstract:**

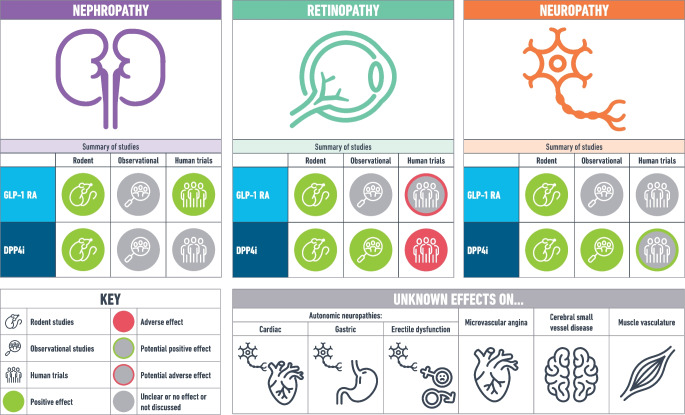

**Supplementary Information:**

The online version contains a slideset of the figures for download, which is available to authorised users at 10.1007/s00125-023-05988-3.

## Introduction

In the last 20 years, several incretin-based therapies, namely glucagon-like peptide-1 (GLP-1) receptor agonists (GLP-1RAs, incretin mimetics) and dipeptidyl peptidase-4 inhibitors (DPP-4is, incretin enhancers), have been developed for people with type 2 diabetes [1]. Although DPP-4is have proven cardiovascular safety [2, 3], only GLP-1RAs have demonstrated a reduction in major adverse cardiac events (MACE) [4, 5]. These findings have come from the need for new glucose-lowering therapies (GLTs) to demonstrate cardiovascular safety prior to approval by the Food and Drug Administration. Whether these therapies reduce the risk of microvascular diseases is less clear, as many of these cardiovascular outcome trials (CVOTs) do not report extensive microvascular outcomes. Furthermore, these trials were not designed to investigate microvascular outcomes, meaning greater statistical uncertainty to detect an impact. This review summarises evidence on whether incretin therapies could reduce microvascular disease, summarising observational studies and clinical trials, and exploring potential mechanisms.

## Microvascular disease and the potential of incretin therapies

**Fig. 1:**
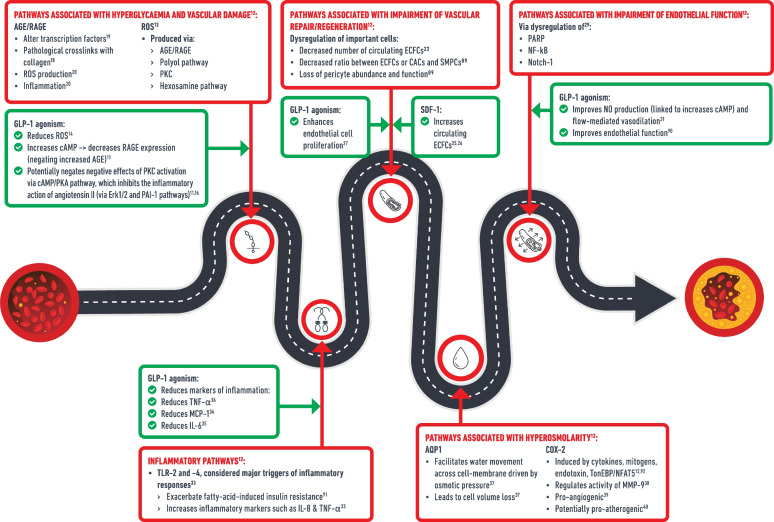
Pathophysiology of microvascular disease in diabetes and protective actions of incretin-based therapies beyond glucose-lowering effects. Based on the processes as summarised by Madonna et al 2017 [1]. AQP, aquaporin; CACs, circulating angiogenic cells; COX, cycloxygenase; ECFC, endothelial colony forming cells; MCP, monocyte chemoattractant protein; MMP, matrix metalloproteinase; PAI, plasminogen activator inhibitor; PKA, protein kinase A; SMPCs, smooth muscle progenitor cells; TLR, toll-like receptor; TonEBP/NFAT5, tonicity-responsive enhancer-binding protein/nuclear factor of activated T cells 5. This figure is available as part of a downloadable slideset

Incretin therapies could play an important role in the prevention of microvascular disease via an increase in GLP-1 agonism. This was initially suggested by bariatric surgery, which increases GLP-1 and is highly efficacious in the primary prevention of microvascular disease (RR 0.37, 95% CI 0.30, 0.46) [6, 7]. Furthermore, GLP-1 agonism increases beta cell preservation and insulin secretion and decreases glucagon secretion, leading to a reduction in plasma glucose [8, 9]. GLP-1 agonism also delays gastric emptying, with subsequent slower digestion of carbohydrate and a reduction in the peak concentration of postprandial glucose [10]. The reduction in hyperglycaemia is likely to attenuate all pathophysiological processes that lead to microvascular disease in diabetes, as this is the ultimate cause of complications, however, GLP-1 agonism may also attenuate specific pathophysiological processes on top of the glucose-lowering effect [11]. These are summarised in Fig. 1, based on the processes summarised by Madonna et al [12].

Hyperglycaemia results in activation of the polyol pathway, which leads to loss of NADPH and increased production of reactive oxygen species (ROS) [13]. ROS production is further increased via generation of AGEs and their interaction with receptors (RAGE); the protein kinase C (PKC) pathway; and the hexosamine pathway. Interestingly, GLP-1 agonism may attenuate some of these processes as its administration is associated with decreased ROS [14, 15]. For example, GLP-1 agonism is associated with increased cellular cAMP and activity of protein kinase A, which potentially offset effects of the PKC pathway [11, 16], and is also associated with decreased RAGE expression [11, 17], which may additionally offset further pathological sequalae, such as the crosslinking of collagen (basement membrane thickening) [18], alteration of transcription factors [19] and inflammation [20].

The microcirculation in type 2 diabetes is associated with an abnormal profile of vascular progenitor cells [21–23], with decreased endothelial colony forming cells (CD34+KDR+ cells identified on flow cytometry) and decreased circulating angiogenic cells, which may adversely impact cardiovascular tissue repair/regeneration and susceptibility to atherogenesis [22, 23]. DPP-4is, in particular, may attenuate this pathway because as well as inhibiting the breakdown of GLP-1, they also inhibit the breakdown of stromal cell-derived factor-1 (SDF-1) (or its commonly measured isoform, SDF-1α) [24]. In vitro research supports a role of SDF-1 in promoting angiogenesis as it is associated with an increase in progenitor cells (identified by their uptake of acetylated LDL [AcLDL]) [25]. Furthermore, this is supported by evidence in human participants as sitagliptin (a DPP-4i) was associated with an increase in circulating CD34+KDR+ cells identified by flow cytometry, compared with control participants [26]. GLP-1 agonism, shown to improve angiogenesis in vitro [27, 28], may also improve vascular repair/regeneration.

Endothelial dysfunction is also observed in diabetes, with impaired nitric oxide (NO) synthesis [21]. Contributing to this is the activation of poly-ADP-ribose polymerase (PARP) in response to diabetic microvascular damage, which triggers an inflammatory cascade via NF-κB activation [12, 29]. Decreased Notch-1 (which inhibits PARP) additionally augments this [29]. Potentially countering this, GLP-1 agonism is associated with improved NO production in vitro and flow-mediated vasodilation in human study participants [30, 31]. Through improved vascular angiogenesis and endothelial function, leading to increased blood flow, insulin sensitivity may also be improved as greater perfusion leads to greater glucose and insulin delivery and greater cellular glucose uptake [32].

Diabetes is also associated with a proinflammatory state, with increased circulating IL-8 and TNF-α [12, 13]. Toll-like receptors-2 and -4 play a central role by activating NF-κB and triggering an inflammatory cascade [33]. GLP-1 agonism ameliorates ROS, which may drive these processes [14, 15]. An anti-inflammatory effect of GLP-1 agonism is further suggested by an associated decrease in TNF-α, monocyte chemoattractant protein-1 and IL-6 [34–36].

Further microvascular damage occurs via hyperosmolarity, driven by hyperglycaemia and the highly osmotic sorbitol (polyol) pathway [12, 13]. Hyperosmolarity induces the expression of Aquaporin-1 channels, which are permeable to water, and consequently result in an adverse loss of intracellular volume [37]. Cyclooxygenase-2 is also triggered by hyperosmolarity, and has pathological pro-angiogenic, and potentially pro-atherogenic, effects [38–40]. It is unknown whether incretins have specific mechanisms to counteract hyperosmolarity-driven damage, but, as with other pathways, they will likely ameliorate this via glucose-lowering effects.

An appreciation that the vasculature differs across end-organs is also required when considering pathology, as the effects of incretins may similarly differ. For example, whilst insulin resistance is important in determining capillary rarefaction and vascular dysfunction in skeletal muscle [32], loss of retinal perfusion is more related to loss of autoregulation and early pericyte and endothelial cell death, amongst other factors [41]. Furthermore, end-stage proliferative retinopathy is driven by neovascularisation [41], which may be less detrimental elsewhere. Converse to reduced blood flow, vasodilation of renal afferent arterioles is seen in early diabetes, which increases glomerular BP and may drive pathological hyperfiltration [42]. Similar differences in both the pathology of complications and mechanisms of impact with incretin therapies may occur within different microvasculatures of relevance to common microvascular complications in diabetes, for example between the blood–brain- and blood–retinal barriers.

In addition to the pathways described in Fig. 1, concomitant obesity, hypertension and dyslipidaemia also contribute to vascular and end-organ damage. GLP-1RAs cause, via an anorexigenic mechanism, a reduction in mean body weight compared with placebo, which varies from −3.80 kg (95% CI −4.46, −3.14) for subcutaneous semaglutide to −0.80 kg (−1.41, −0.19) for dulaglutide [43]. Similarly, GLP-1RAs result in mean systolic BP reduction, from −1.76 mmHg (95% CI −2.82, −0.70) for exenatide extended release to −3.06 mmHg (−4.21, −1.91) for oral semaglutide, and, although to a lesser extent, several GLP-1RAs result in a diastolic BP reduction [43]. GLP-1RAs also decrease mean LDL-cholesterol (ranging from −0.08 to −0.16 mmol/l) and some show a modest decrease in triacylglycerol (for liraglutide: −0.30 mmol/l [95% CI −0.49, −0.11]) compared with placebo [44].

In addition to vascular effects, GLP-1 receptors have also been identified in the peripheral nervous system and kidneys, where they may have direct actions on organ-specific pathophysiological processes [45–47]. Similarly, SDF-1 may have beneficial end-organ effects, particularly for neuropathy, via enhancing tissue repair [48]. However, these benefits are debated [24, 46], with potential harms to the retina reported [49], and conflicting nephropathy findings [24, 46]. DPP-4 may also regulate other hormones, including granulocyte-macrophage colony-stimulating factor and IL-3 [50], but the significance of this for microvascular disease is unknown.

## Nephropathy

### GLP-1RAs

Evidence of a nephroprotective effect of GLP-1RAs comes from several rodent studies demonstrating reduced albuminuria, oxidative stress and inflammation; improved BP; and fewer pathological histology findings (glomerular hypertrophy, mesangial matrix expansion and glomerular lipid accumulation) [11, 51].

Data from CVOTs for GLP-1RAs are harder to interpret as they were not designed for renal outcomes. Hard renal outcomes (e.g. end-stage renal failure, dialysis or renal death) have a much lower incidence than MACE, for which these trials are powered to detect a difference; this could mean that even a very large difference may not be detected due to the lower absolute risk and subsequent larger statistical uncertainty. Therefore, unsurprisingly, individual CVOTs do not find a reduction in the risk of hard renal endpoints. However, trials do show a consistent reduction in albuminuria compared with placebo (RR 0.77, 95% CI 0.70, 0.84 for new macroalbuminuria in a meta-analysis) [52]. Similarly, meta-analysis demonstrated a reduction in a composite renal outcome that included albuminuria [53], but it remained unclear whether this simply reflected a large risk reduction in albuminuria rather than a reduction in other renal outcomes. However, meta-analysis has now confirmed a risk reduction for a renal composite outcome that excluded albuminuria with GLP-1RAs (RR 0.92, 95% CI 0.84, 0.99) [52], although of a lesser magnitude compared with albuminuria. The FLOW trial (semaglutide vs placebo), an RCT powered for a composite renal outcome that excludes albuminuria, is underway to clarify this [54].

The mechanisms behind the renoprotective effects of GLP-1 therapy are likely to be multifactorial, and GLP-1 receptors within the kidney may play a crucial role. One meta-analysis by Chalmoukou et al showed that the reduction in nephropathy was strongly explained by systolic BP reduction [52], whereas another found that the risk reduction was explained by HbA_1c_ reduction (not found in the meta-analysis by Chalmoukou et al) [53]. Interestingly, despite the impressive reduction in BMI with GLP-1RA use, this was not a mediating factor. Analysis of individual-level data is needed alongside further mechanistic work to clarify these findings.

### DPP-4is

As with GLP-1RAs, early rodent studies were promising for nephroprotective effects of DPP-4is, with observed improvements in albuminuria, filtration barrier remodelling, glomerular oxidative stress, creatine clearance and histological markers [11, 51]. CVOTs similarly showed a reduction in albuminuria, although to a lesser extent than with GLP-1RAs, with less albuminuria progression (HR=0.86 [95% CI 0.78, 0.95]) in the CARMELINA trial (linagliptin) [55], and similar benefits in the SAVOR-TIMI 53 trial (saxagliptin) [56]. One observational study showed a further beneficial association of DPP-4is compared with sulfonylureas, with a composite renal outcome that did not include albuminuria (HR=0.91 [95% CI 0.85, 0.97]) [57]. However, this has not translated to a decrease in the composite renal outcome in a meta-analysis of RCTs [52]. Given the reasonably narrow confidence intervals (RR=1.03 [95% CI 0.93, 1.15]), it is unlikely that this is related to lack of power and suggests a true lack of substantial benefit.

### Why GLP-1RAs may be more reno-protective than DPP-4is

Collectively, whilst both GLP-1RAs and DPP-4is appear to improve albuminuria, only GLP-1RAs result in an improvement in the more clinically meaningful composite renal outcome. There are two hypotheses for why this difference exists. First, GLP-1 agonism has numerous positive physiological effects (Fig. 1) and GLP-1RAs, as exogenous incretin mimetics, result in a far higher degree of GLP-1 agonism compared with DPP-4is, which are incretin enhancers and limited to increasing endogenous production of GLP-1 [58]. Furthermore, some of the benefits of GLP-1 agonism could be offset with DPP-4is through the increase in SDF-1, with potential associations with pathological processes such as natriuresis and renal hyperfiltration, atherogenesis, podocyte injury and glomerulosclerosis [24], although this is disputed [46]. Future RCTs involving DPP-4is could measure changes in plasma SDF-1 concentrations to investigate if this is associated with outcomes.

## Retinopathy

### GLP-1RAs

As with nephropathy, rodent studies suggested protective effects of GLP-1 agonism for retinopathy, including decreased glial activation, neural apoptosis and electroretinographical abnormalities; protection of the blood–retinal barrier; downregulation of growth factors; and prevention of cell loss in the inner and outer nuclear layers (albeit transiently) [11, 59]. Furthermore, these beneficial processes may occur through glucose-independent pathways, including reduced retinal glutamate production and increased prosurvival signalling pathways [11, 59].

Unfortunately, RCTs suggest a different picture to pre-clinical studies, and concern has arisen because semaglutide was associated with an increased risk of retinopathy complications in the SUSTAIN-6 RCT (HR 1.76, 95% CI 1.11, 2.78) [4]. Other CVOTs have not found significant differences in ocular outcomes (although they were not powered for this). Furthermore, most meta-analyses of RCTs do not find significant associations between GLP-1RAs and retinopathy outcomes [60–62], however, there were wide confidence intervals, contributed to by heterogeneity in findings. In two meta-analyses, GLP-1RAs were associated with retinopathy progression, but both had flaws. In one, authors only included trials with cardiovascular benefit, but excluded studies utilising off-market drugs and included a trial (PIONEER-6) where superiority of the primary cardiovascular outcome was not shown [63, 64]. After changing the included studies accordingly, there was no longer an association with retinopathy [64]. The other positive meta-analysis was contributed to by a mistaken input for the LEADER trial [65]. One meta-analysis looking at semaglutide use only, found an increased risk of retinopathy compared with placebo [66].

There has been much debate as to why retinopathy risk was higher in the SUSTAIN-6 trial. First, participants in SUSTAIN-6 had a high prevalence of background retinopathy, and post hoc analysis suggested that in individuals without pre-existing retinopathy, ocular events were no different [67]. As such, it may be that these adverse outcomes are limited to those at high baseline risk. Furthermore, compared with other CVOTs, participants in SUSTAIN-6 had a high baseline HbA_1c_, owing to no upper limit for HbA_1c_ in the inclusion criteria, and a subsequent large reduction in HbA_1c_ (larger still in the subgroup with pre-existing retinopathy) [60, 67]. The increased risk may be related to the rapid decrease in HbA_1c_ that occurs with GLP-1RA use; this is supported by meta-analysis showing that the magnitude of HbA_1c_ reduction is associated with the risk of retinopathy outcomes with GLP-1RA use (and not with systolic BP or weight) [60]. This phenomenon has been noted before, such as within the DCCT where intensive treatment was associated with both a larger HbA_1c_ reduction from baseline and an increased risk of worsening of retinopathy at 6 and/or 12 months [68]. Interestingly, over a mean 6.5 years follow-up, the overall risk of retinopathy and progression of retinopathy was reduced with intensive therapy compared to conventional therapy [69]. Given that SUSTAIN-6 had an observation period of approximately 2 years, it is unclear whether a longer follow-up may have seen a reversal of the negative relationship between semaglutide and retinopathy complications. The FOCUS trial, an ongoing RCT comparing semaglutide and placebo, will investigate this further and will report retinopathy progression at 5 years as the primary outcome [70].

### DPP-4is

Pre-clinical studies showed similar promise that DPP-4is may reduce the risk of retinopathy. In rodent studies, DPP-4 inhibition has been shown to prevent blood–retinal barrier breakdown, decrease retinal inflammation and neuronal apoptosis, reduce gene expression responsible for increased levels of growth factors and decrease neovascularisation [11, 59]. Moreover, there is supportive observational evidence in humans. In a retrospective cohort study of German electronic medical records (*N*=630 after propensity score matching), vildagliptin was associated with a lower incidence of retinopathy (OR 0.55, 95% CI 0.39, 0.77) [71]. This was supported by another smaller retrospective observational study (*N*=82) in South Korea finding that DPP-4is were associated with reduced progression of retinopathy [72].

Despite these positive findings, interventional studies suggest a different picture: DPP-4is may increase risk of diabetic retinopathy. In the TECOS trial, there was a higher crude prevalence of diabetic eye disease (3.1% in the sitagliptin group vs 2.5% in placebo) and retinopathy (2.8% with sitagliptin vs 2.2% with placebo) [73]. Although not reported, these differences correspond to an OR of 1.26 (95% CI 1.04, 1.54) for diabetic eye disease and 1.31 (1.06, 1.61) for retinopathy. Meta-analysis of RCTs supports that DPP-4i use is associated with increased risk of diabetic retinopathy in pairwise meta-analysis (OR 1.27, 95% CI 1.05, 1.53) [74]. Similar to the increased retinopathy risk with GLP-1RA use, this may be related to the reduction in HbA_1c_ with DPP-4is (mean reduction: 0.30% to 0.80%), as across GLTs retinopathy risk is related to the magnitude of HbA_1c_ decrease [74].

### Lack of retinal protection with incretin therapies

Given the data, caution should be given to the use of semaglutide and DPP-4is with regards to risk of retinopathy. Debate exists as to why the protective effects of incretin therapies were never realised in CVOTs. This could be related to the physiological differences between rodents and humans, or to the lack of GLP-1 receptors within the retina, in contrast to the kidneys and nerves [30]. For DPP-4is, the additional increase in SDF-1 may be harmful, due to its neovascular effect that may cause proliferation and damage that may mimic the pathological processes that occur in the development of diabetic retinopathy [49].

## Peripheral neuropathy

### GLP-1RAs

Early pre-clinical diabetic rodent experiments demonstrated various improvements in nerve function following initiation of a GLP-1RA. These benefits included improvement of sensory and motor nerve electrophysiology and behavioural sensory loss, reduction of intraepidermal nerve fibre densities in the sole skins, restoration of myelin fibre size, prevention of Schwann cell apoptosis, reduction of myelinated nerve fibre density and reduction of neuropathic pain [75].

Whether GLP-1RAs result in a clinically significant decrease in neuropathy incidence or severity remains to be seen, with only a few studies in humans undertaken, most of which involved less than 100 participants (Table 1). In the two larger studies, an observational study from the United States national claims database OptumLabs Data Warehouse (*N*=8252) and the GRADE RCT (*N*=5047), no difference in incidence of neuropathy was seen compared with other GLTs [76, 77].

**Table 1:** The role of incretins in the prevention of peripheral neuropathy, a summary of research in humans

Level of evidence	Reference	Key characteristics	Key findings
GLP-1RAs			
Cohort studies	Issar et al 2021 [93]	*N*=90. Exenatide vs DPP-4i vs SGLT2i vs healthy control participants for cross-sectional study. Ten patients taking exenatide followed for a further 3 months in a prospective study and compared with 32 control participants.	Improvements in nerve physiological measures with exenatide use compared with no exenatide use, including improved nerve function, S2 accommodation, superexcitabillity and subexcitabilty. These changes were independent of HbA_1c_ reduction.
	Deng et al 2022 [76]	*N*=8252. Compared insulin glargine, liraglutide, glimepiride and sitagliptin. Propensity scores used to account for differences in baseline characteristic. These were created from several baseline characteristics (including HbA_1c_, ethnicity and comorbidity).	No significant difference in neuropathy incidence with liraglutide use compared with other medications.
RCTs	Ponirakis et al 2020 [94]	*N*=38. Three months of pioglitazone and exenatide vs glargine with aspart insulin.	Vibration perception worsened after 3 months’ usage of pioglitazone and exenatide with no change in neuropathic pain.
	Jaiswal et al 2015 [95]	*N*=46. Exenatide vs insulin glargine.	No significant difference in incident neuropathy or electrophysiology markers.
	Brock et al 2019 [96]	*N*=36 (type 1 diabetes) with confirmed polyneuropathy. Randomised to liraglutide or placebo.	No effect of liraglutide on measures of nerve function.
	GRADE Study Research Group et al 2022 [77]	*N*=5047. Compared insulin glargine, liraglutide, glimepiride and sitagliptin.	No difference in incidence of diabetic polyneuropathy between any of the four medications over a median follow-up of 5 years.
Simulated long-term follow-up of RCT	Sullivan et al 2009 [97]	*N*=746 + three *n*=5000 hypothetical cohorts. Liraglutide vs glimepiride.	Predicted neuropathy incidence was lower in the liraglutide group.
DPP-4is			
Cross-sectional studies	Issar et al 2021 [93]	*N*=90. Exenatide vs DPP-4i vs SGLT2i vs healthy control participants.	Physiological markers of nerve function were similar in DPP-4i and SGLT2i groups with more favourable findings in the exenatide group.
Cohort studies	Kolaczynski et al 2016 [71]	*N*=16,321. Propensity score matching to compare vildagliptin use to sulfonylureas. Propensity scores were derived from the probability of treatment assignment from the following baseline factors: age, sex, line of therapy, HbA_1c_ score, duration of disease (<5 years vs ≥5 years), duration of treatment, previous hypoglycaemic events, co-prescribed medications and number of comorbidities.	Lower neuropathy incidence with vildagliptin OR 0.71 (95% CI 0.60, 0.85).
	Deng et al 2022 [76]	*N*=8252. Compared insulin glargine, liraglutide, glimepiride and sitagliptin. Propensity scores used to account for differences in baseline characteristics. These were created from several baseline characteristics (including HbA_1c_, ethnicity and comorbidity).	Sitagliptin appeared to be associated with lower neuropathy incidence compared with glimepiride HR 0.87 (95% CI 0.76, 0.99), although this was not statistically significant after the *p* value was adjusted for multiple testing (*p*=0.09).
RCTs	da Silva et al 2015 [98] (abstract only)	*N*=30. Sitagliptin vs NPH insulin in patients with type 2 diabetes of long duration.	No difference in electrophysiological markers of neuropathy at 1 year.
	TECOS trial, original publication: Green et al 2015 [73]	*N*= 14,671. Sitagliptin vs placebo.	Incidence of diabetic neuropathy 4.1% in Sitagliptin group, 3.8% in placebo.
	TECOS trial, CKD participants only: Engel et al 2017 [99]	*N*=3324. Sitagliptin vs placebo. Subgroup analysis of patients with CKD.	No significant difference in neuropathy incidence was observed, risk difference for sitagliptin vs placebo using M-N method = 0.21 (95% CI −1.09, 1.53).
	GRADE Study Research Group et al 2022 [77]	*N*=5047. Compared insulin glargine, liraglutide, glimepiride and sitagliptin.	No difference in incidence of diabetic polyneuropathy between any of the four medications over a median follow-up of 5 years.
	Gabriel et al 2023 [79]	*N*=658. Linagliptin vs placebo vs metformin vs linagliptin + metformin.	Linagliptin associated with 19.5% (95% CI 10.1, 29.0) reduction in small fibre peripheral neuropathy compared with placebo.

CKD, chronic kidney disease; SGLT2i, sodium–glucose cotransporter 2 inhibitor

### DPP-4is

Similar to rodent studies with GLP-1RAs, those with DPP-4is showed promise in reducing the incidence of neuropathy, with observations including decreased nerve fibre density and improved nerve conduction velocity [78]. Evidence from human studies is suggestive of a beneficial effect (Table 1). Two large observational studies of electronic medical records suggested a decreased incidence of neuropathy with both vildagliptin and sitagliptin compared to sulfonylureas [71, 76]. Findings from large RCTs are mixed, however, with only one trial suggesting a 19.5% (95% CI 10.1, 29.0) reduction in small fibre peripheral neuropathy with linagliptin compared to placebo [79]. It is unclear why these findings differ, but it may be related to different comparators (placebo or other GLTs), different DPP-4is, different measures of neuropathy and varying statistical power.

### Comparing GLP-1RAs and DPP-4is for peripheral neuropathy

Although evidence is inconclusive, DPP-4is appear to be more promising than GLP-1RAs in reducing peripheral neuropathy incidence. This is further supported by an observational study showing that liraglutide appeared to be associated with a higher incidence of neuropathy compared with sitagliptin (HR 1.36 [95% CI 1.03, 1.80]). However, this was not significant when accounting for multiple testing (*p*=0.09) [76]. A potential reason for a theoretical superiority of DPP-4is over GLP-1RAs may be because of the additional increase in SDF-1 [48].

## Autonomic neuropathy

Given that some evidence exists that incretin therapies may decrease peripheral neuropathy risk, particularly within rodent studies, it follows that they may additionally protect against disorders of the autonomic nervous system via the same mechanisms, including cardiac autonomic neuropathy (CAN), gastroparesis and erectile dysfunction.

Whilst cardiovascular safety for DPP-4is and GLP-1RAs is clear [2–5], measures of CAN are strong predictors of cardiovascular death and can also cause symptoms, most notably from orthostatic hypotension. It is unclear how GLP-1 agonism affects the autonomic nervous system and these may be more complex than the mechanisms related to peripheral neuropathy due to the many inputs that determine both sympathetic and parasympathetic autonomic activity [80]. Furthermore, the role of GLP-1RAs on sympathetic drive is particularly debated. For example, GLP-1 receptors have been observed in the carotid body and activation of these diminish the sympathetic response to high plasma glucose and/or insulin [81]. Conversely, GLP-1RAs cause an increased resting heart rate (increased by approximately 3 beats-per-min), which may suggest an increase in sympathetic drive [80]. Other studies have reported decreased vagal tone, decreased 30:15 value from the lying-to-standing test and a decreased heart rate variability with GLP-1 RAs [47, 80], However, there are inconsistencies in the literature. Although unclear at present, this evidence suggests that GLP-1RAs may lead to autonomic imbalance and CAN.

Determining whether incretins may be beneficial for reducing autonomic neuropathy that contributes to gastroparesis is difficult, namely because GLP-1 receptor activation in the stomach has a potent effect at delaying gastric emptying [82]. Despite this, glucose-lowering effects may protect against a neuropathy-induced gastroparesis, as long-term glucose control is associated with lower incidence of gastroparesis in the follow-up of the DCCT/EPIC trials [83], in addition to the other discussed mechanisms by which incretin therapies may protect nerves and the microvasculature.

Erectile function depends on healthy vascular and nervous function; disorders of either system can result in erectile dysfunction [84]. There are therefore two pathophysiological processes by which diabetes can result in erectile dysfunction and that incretin therapies may modify. Few data exist, although a positive effect was suggested in the REWIND RCT of dulaglutide, with a small reduction in erectile dysfunction incidence and severity vs placebo [85].

## Incretins and other microvascular pathologies

Vascular damage may also have a direct causative effect in other microvascular pathologies, such as microvascular cardiac angina and cerebral small vessel disease (cSVD), and may have further clinical significance in determining microvascular flow reserve in skeletal muscle. The effect of incretin therapies on these outcomes has not been well studied in humans thus far.

Type 2 diabetes is a recognised risk factor for microvascular cardiac angina, which is principally considered a disorder of endothelial function and a subsequent impairment in the control of coronary blood flow, leading to hypoxia and chest pain [21]. Given that GLP-1 has been shown in several studies to improve endothelial function and flow-mediated vasodilation [27, 28, 30], research should investigate whether incretins may be beneficial for the prevention or management of microvascular angina.

Similarly, cSVD is associated with type 2 diabetes as well as cognitive decline, dementia and lacunar stroke [86]. However, the pathophysiological mechanisms may differ to microvascular cardiac angina, as it is related to inflammation, blood–brain barrier disruption and vascular remodelling. Again, as incretin therapies are associated with decreased inflammation and ROS production [11, 14, 15], these may also have a role in the prevention of cSVD.

Skeletal muscle microvascular rarefaction and subsequent reduction in capillary blood flow is also associated with type 2 diabetes [87, 88]. This is likely to have important implications for cardiovascular fitness and response to exercise, both features of health status. It may also impair the endocrine function of skeletal muscle in glucose regulation in a theoretical negative cycle: less skeletal muscle blood flow, less glucose uptake in response to insulin, higher plasma glucose, further microvascular damage [87, 88]. In rodent studies, GLP-1 agonism has already been demonstrated to increase capillary density and improves features of insulin resistance in skeletal muscle, with evidence of improved skeletal muscle blood flow in humans with diabetes [32]. Given the benefits of GLP-1 to endothelial function and angiogenesis [27, 28, 30], further research into skeletal muscle microvascular disease and incretin therapies is warranted in humans.

## Conclusion

**Fig. 2:**
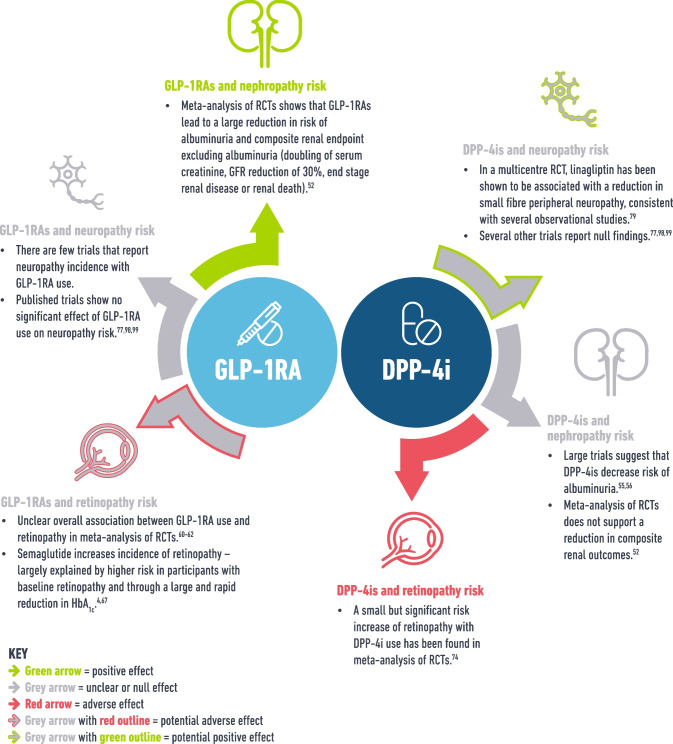
A summary of the observational and trial evidence for incretin-based therapies and microvascular diseases. This figure is available as part of a downloadable slideset

In conclusion, incretin therapies differ in their effect on microvascular disease by drug type (GLP-1RA vs DPP-4i) and by microvascular outcome (Fig. 2). High quality evidence demonstrates that GLP-1RAs reduce risk of adverse renal outcomes whereas DPP-4is do not. It remains unclear whether GLP-1RAs may cause adverse ocular events, but use in patients with background retinopathy, high HbA_1c_ or the use of semaglutide specifically, may carry higher risk and caution is advised. Adequately powered RCTs of long duration are required to clarify this which should report and investigate known risk factors for retinopathy complications (e.g. background retinopathy, diabetes duration, beta cell function) as potential confounders/mediators. Clinical caution with DPP-4is and retinopathy is similarly warranted. There is little evidence that GLP-1RAs reduce the incidence of neuropathic pathologies (peripheral neuropathy, CAN, gastroparesis and erectile dysfunction) in interventional trials, despite promising pre-clinical research. Similarly, the relationship with DPP-4is and neuropathy remains unclear, although it appears to be more promising for peripheral neuropathy. Again, adequately powered trials and real-world monitoring are needed, and ideally all trials should report peripheral neuropathy incidence (alongside less studied microvascular outcomes) to enable meta-analysis and improve understanding. Clearly, the mechanisms by which incretins exert their protective/adverse effects on microvascular outcomes are likely to be multifactorial, but they remain poorly understood and further mechanistic work is needed.

## Supplementary Information

Below is the link to the electronic supplementary material.

Supplementary file1 (PPTX 853 KB)

## Abbreviations

CAN: Cardiac autonomic neuropathy
cSVD: Cerebral small vessel disease
CVOTs: Cardiovascular outcome trials
DPP-4i: Dipeptidyl peptidase-4 inhibitor
GLP-1: Glucagon-like peptide-1
GLP-1RA: Glucagon-like peptide-1 receptor agonist
GLTs: Glucose-lowering therapies
MACE: Major adverse cardiac events
NO: Nitric oxide
PARP: Poly-ADP-ribose polymerase
PKC: Protein kinase C
RAGE: Receptors for advanced glycation end-products
ROS: Reactive oxygen species
SDF-1: Stromal cell-derived factor-1
