# Supplementary material for: Incretins and microvascular complications of diabetes: neuropathy, nephropathy, retinopathy and microangiopathy
Source: Diabetologia. 2023 Aug 19;66(10):1832–45. doi: 10.1007/s00125-023-05988-3 (PMC10474214; doi:10.1007/s00125-023-05988-3)
Supplement: Supplementary file 1 — Supplementary file1 (PPTX 853 KB) [file 125_2023_5988_MOESM1_ESM.pptx]

## Slide 1
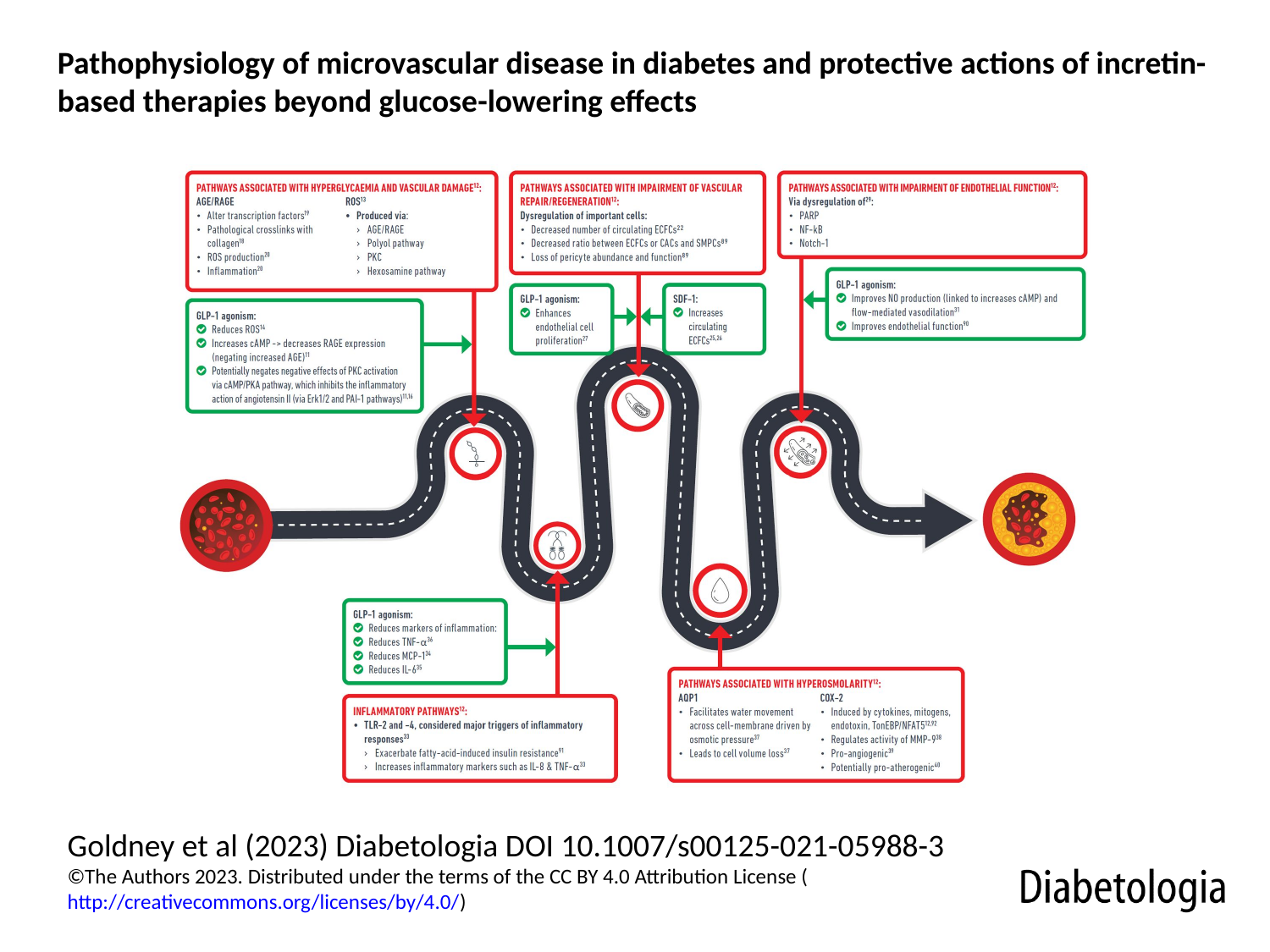

Pathophysiology of microvascular disease in diabetes and protective actions of incretin-based therapies beyond glucose-lowering effects
Goldney et al (2023) Diabetologia DOI 10.1007/s00125-021-05988-3
©The Authors 2023. Distributed under the terms of the CC BY 4.0 Attribution License (http://creativecommons.org/licenses/by/4.0/)

## Slide 2
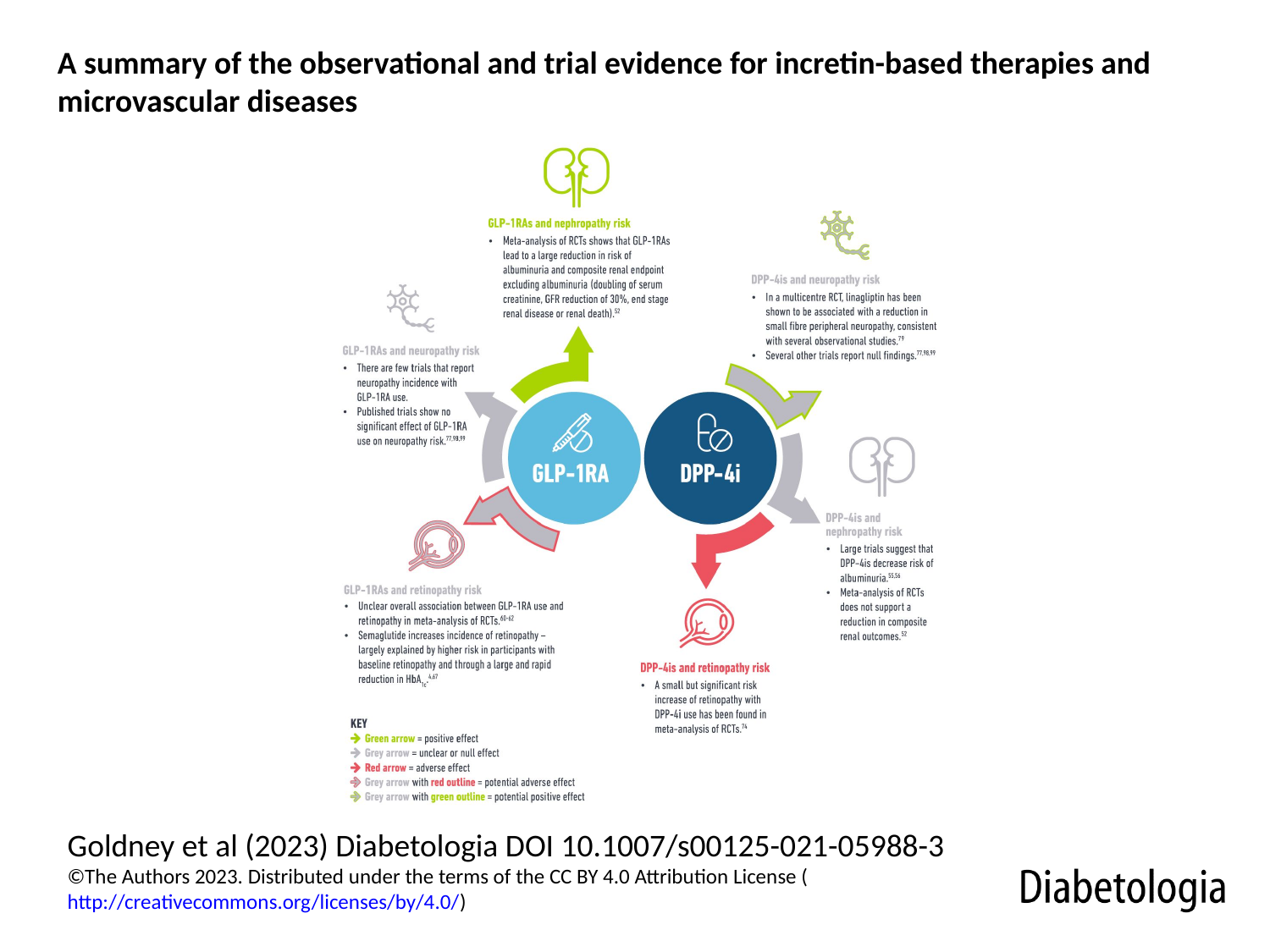

A summary of the observational and trial evidence for incretin-based therapies and microvascular diseases
Goldney et al (2023) Diabetologia DOI 10.1007/s00125-021-05988-3
©The Authors 2023. Distributed under the terms of the CC BY 4.0 Attribution License (http://creativecommons.org/licenses/by/4.0/)
